# Combined Administration of *Lactobacillus* or *Bifidobacterium* Offers Enhanced Antidepressant and Anxiolytic Activity in a Dose Dependent Manner

**DOI:** 10.1002/brb3.70564

**Published:** 2025-05-18

**Authors:** Aliu Zanzeh Bankah, Thomas Amatey Tagoe, Emmanuel Darko, Righteous Agoha, Elmer Nayra Ametefe, Kennedy Kwami Edem Kukuia, Samuel Adjei

**Affiliations:** ^1^ Department of Physiology University of Ghana Legon Ghana; ^2^ Department of Biochemistry, Cell and Molecular Biology University of Ghana Legon Ghana; ^3^ Department of Animal Experimentation, Noguchi Memorial Institute for Medical Research University of Ghana Legon Ghana; ^4^ Department of Medical Pharmacology University of Ghana Legon Ghana

**Keywords:** gut microbiome, lactic acid bacteria, mental health, probiotics

## Abstract

**Purpose:**

Gut microbiota is strongly linked to the activity of the bidirectional gut‐brain axis, which influences neuropsychological processes at multiple levels. Changes in the gut microbiota have been implicated in mood disorders, and probiotics have been explored for their ability to mitigate the effects of stress on mental health. Here, we investigated the therapeutic benefits of different concentrations and combinations of *Lactobacillus and Bifidobacterium* in a mouse model of stress induced depression and anxiety.

**Methods:**

Sixty‐three male ICR mice (6–8 weeks old; 20–25g) divided into nine groups were used for this study. The test groups underwent chronic unpredictable mild stress protocols for two weeks before receiving low (10^4^ CFU/ml) or high (10^8^ CFU/ml) doses of either monotherapy (*Lactobacillus* or *Bifidobacterium*) or a combination therapy (*Lactobacillus* and *Bifidobacterium*) for four weeks. The antidepressant, fluoxetine, served as the positive control. Measurements of weight and sucrose preference were performed at four time points in addition to a battery of behavioral tests (open field tests, forced swim test, tail suspension test, and hot plate test) at the endpoint to assess depression and anxiety‐like behavior.

**Results:**

Low doses of the probiotic formulation (mono‐ or combined therapy) reversed weight loss but not anhedonia. In contrast, high doses of probiotic formulations (mono‐ or combined therapy), along with fluoxetine, were effective in reversing the weight loss and anhedonia caused by chronic unpredictable mild stress. Probiotics ameliorated stress‐induced immobility as measured by both the forced swim and tail suspension tests, while also reducing anxiety‐like behavior (increased peripheral activity) in the open field test. High doses of mono‐ or combined therapy increased curling behavior in the tail suspension test, whereas fluoxetine failed to do so.

**Conclusion:**

This study indicates the species‐ and dose‐dependent beneficial effects of probiotics on behavioral outcomes associated with depression while also reversing weight loss. Evidence suggests that probiotics and fluoxetine may exert antidepressant activity via different mechanisms.

## Graphical abstract

The probiotics *lactobacillus* and *bifidobacterial* exhibit dose‐dependent beneficial effects on anthropometric, behavioral and physiological deficits associated with depression in an animal model. These effects were to a similar degree as fluoxetine, a selective serotonin reuptake inhibitor.

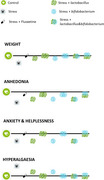

## 1 Introduction

Stress can be described as a response to physical and emotional demands, with the term ‘stressed’ being used when the demands of the situation exceed all available resources (Sharma 2018). Over the years, stress has become a subject of intense study, as it has been implicated in the etiology of multiple dysfunctional states affecting physiological systems, including the digestive and nervous systems (Shukkoor et al. 2016; Steinmetz et al. 2024). For example, stress can impair the function of the intestinal barrier, resulting in increased intestinal permeability (Rudzki and Szulc 2018). Increased intestinal permeability may result in subsequent immunological activation in the gut, affecting the central nervous system via the gut‐brain axis (Jiang et al. 2015; Wu et al. 2022; Xiong et al. 2023). This particular pathway, as described, has been proposed as a possible pathophysiological mechanism in neurological disorders, including anxiety and depression (Carabotti et al. 2015; Rudzki and Szulc 2018). This is supported by multiple studies showing that the gastrointestinal microbiota produces and delivers neuroactive chemicals that operate via the gut–brain axis (Carabotti et al. 2015; Evrensel and Ceylan 2015). Thus, there is a direct link between stress, the gut, and mental health (Xiong et al. 2023).

Animal studies have shown that uncontrollable stressors can alter the gut microbiota and result in depressive behavioral states (Farhan et al. 2014; Zhu et al. 2014; Xiong et al. 2023 **)**. Clinical studies have confirmed this by showing that depression can be triggered by chronic stress, which causes inflammatory cytokines to be released in susceptible individuals (Kiecolt‐Glaser et al. 2015; Madison and Kiecolt‐Glaser 2019). Based on these results, probiotics have been investigated as an alternative therapeutic approach in the management of depression due to the potential to modulate key neurotransmitters via the same gut‐brain axis (Bravo et al. 2011; Desbonnet et al. 2010; Chudzik et al. 2021; O'Mahony et al. 2015; Snigdha et al. 2022).

In this regard, *Lactobacillus* and *Bifidobacterium* have been studied extensively. In mouse models of necrotizing enterocolitis, bifidobacteria have been shown to improve barrier function by stabilizing occludin at tight junctions and claudins 2 and 4 (Di Vincenzo et al. 2024). Additionally, they have antioxidant properties and play a role in immune cell maturation, stimulation of IgA secretion, creation of anti‐inflammatory cytokines, and preservation of intestinal microvillus integrity (Ruiz et al. 2017). Several Lactobacillus species inhibit barrier disruption by upregulating tight junction proteins (Di Vincenzo et al. 2024). Specifically, administration of *Lactobacillus rhamnosus* has been found to reduce stress‐induced increases in corticosterone levels and depression‐related behavior by influencing GABA receptor mRNA expression in specific brain regions (Bravo et al. 2011). Additionally, various Lactobacillus species have been shown to produce neuroactive substances, such as serotonin and dopamine, which may counteract the reduced activity of these neurotransmitters observed in depression (Winter et al. 2018; (Wu et al. 2022; Xiong et al. 2023; Barrett et al. 2012). Clinical studies have also reported a decrease in psychological stress and depression in healthy individuals administered *Lactobacillus helveticus* and *Bifidobacterium longum* (Messaoudi et al. 2011). Overall, these findings highlight the potential of probiotics to mitigate depressive symptoms, both in preclinical and clinical studies.

Despite the potential benefits of probiotics, questions remain regarding the most effective therapeutic doses and combinations of strains. Therefore, this study was designed to ascertain the differences in the therapeutic potential of probiotics as antidepressants and anxiolytics based on various concentrations and strain combinations in an animal model of depression.

## 2 Materials and Methods

### 2.1 Experimental Animals

Sixty‐three Institute of Cancer Research (ICR) mice, aged 6–8 weeks at the start of the study and weighing 20–25 g, were purchased from the Noguchi Memorial Institute for Medical Research. All mice were housed in standard polycarbonate cages (34 × 47 × 18 cm) with soft‐wood shavings as bedding. Normal commercial rodent chow (Agricare, Kumasi—Ghana) and water were available ad libitum throughout the experimental period. All animals were handled in accordance with the Guide for the Care and Use of Laboratory Animals, and the protocols were approved by the College of Health Sciences Ethical and Protocol Review Committee, University of Ghana (**CHS‐Et/M.8.P5.10/2021‐2022**).

### 2.2 Probiotics/Preparation of Bacterial Strains

Lactobacillus and Bifidobacterium spp. (Bifidobacterium bifidum, Lactobacillus plantarum, Lactobacillus salivarius, Lactobacillus casei, Lactobacillus paracasei, Lactobacillus rhamnosus, Bifidobacterium breve, and Bifidobacterium longum) isolated from a commercial product (NOW Probiotic‐10TM, NOW FOODS, Bloomingdale, USA) were used in this study. Selective growth media was used to isolate strains that were confirmed by cell morphology, gram reaction, and catalase tests.

The cultures were incubated overnight at 37°C in MRS broth (for *Lactobacillus* formulations), MRS broth + 0.5% cysteine (for mixed formulations), or MRS broth + 0.5% cysteine (for *bifidobacteria* formulations). After incubation, bacterial pellets were harvested by centrifugation at 2,000 × g for 10 min, washed three times with sterile phosphate‐buffered saline (pH 7.3), and resuspended in distilled water to reach a final concentration of 10^8^ CFU/ml or 10^4^ CFU/ml for the high and low formulations, respectively. The concentration of each culture was determined based on the viable count.

$$\mathrm{CFU}/\mathrm{ml}=\frac{\left(\mathrm{Number}\;\mathrm{of}\;\mathrm{bacterial}\;\mathrm{colonies}\;\mathrm{counted}\;\mathrm{on}\;\mathrm{plate}\;\times \mathrm{Dilution}\;\mathrm{Factor}\right)}{\mathrm{Volume}\;\mathrm{of}\;\mathrm{culture}\;\mathrm{plate}}$$

To exert health benefits, probiotic bacteria must remain viable and survive harsh conditions in the GI tract, with a minimum count of 10^6^ CFU g^−1^ (Nagpal et al., 2012). Based on this premise, high concentrations were chosen at two points above this threshold (10^8^ CFU/ml), and low concentrations were chosen at two points below (10^4^ CFU/ml).

### 2.3 Experimental Animal Groups and Treatment

Animals were individually identified and randomly divided into nine groups (n = 7): Veh‐NS (unstressed), Veh‐S (stressed), Flx (stressed + fluoxetine—10 mg/kg daily: (Sigma‐Aldrich, St. Louis, MO, USA), L.Lacto (stressed + 10^4^ CFU/ml *Lactobacillus* daily), H.Lacto (stressed + 10^8^ CFU/ml *Lactobacillus* daily), L.Bifido (stressed + 10^4^ CFU/ml *Bifidobacterium* daily), H.Bifido (stressed + 10^8^ CFU/ml *Bifidobacterium* daily), L.Mix (stressed + 10^4^ CFU/ml mixed formulation daily) and H.Mix (stressed + 10^8^ CFU/ml mixed formulation daily). All mice were orally administered with 0.02 mL/g of the assigned probiotics within the same time window (9:00am–11:00am).

### 2.4 Establishment of Chronic Unpredictable Mild Stress Mice Model

**FIGURE 1:**
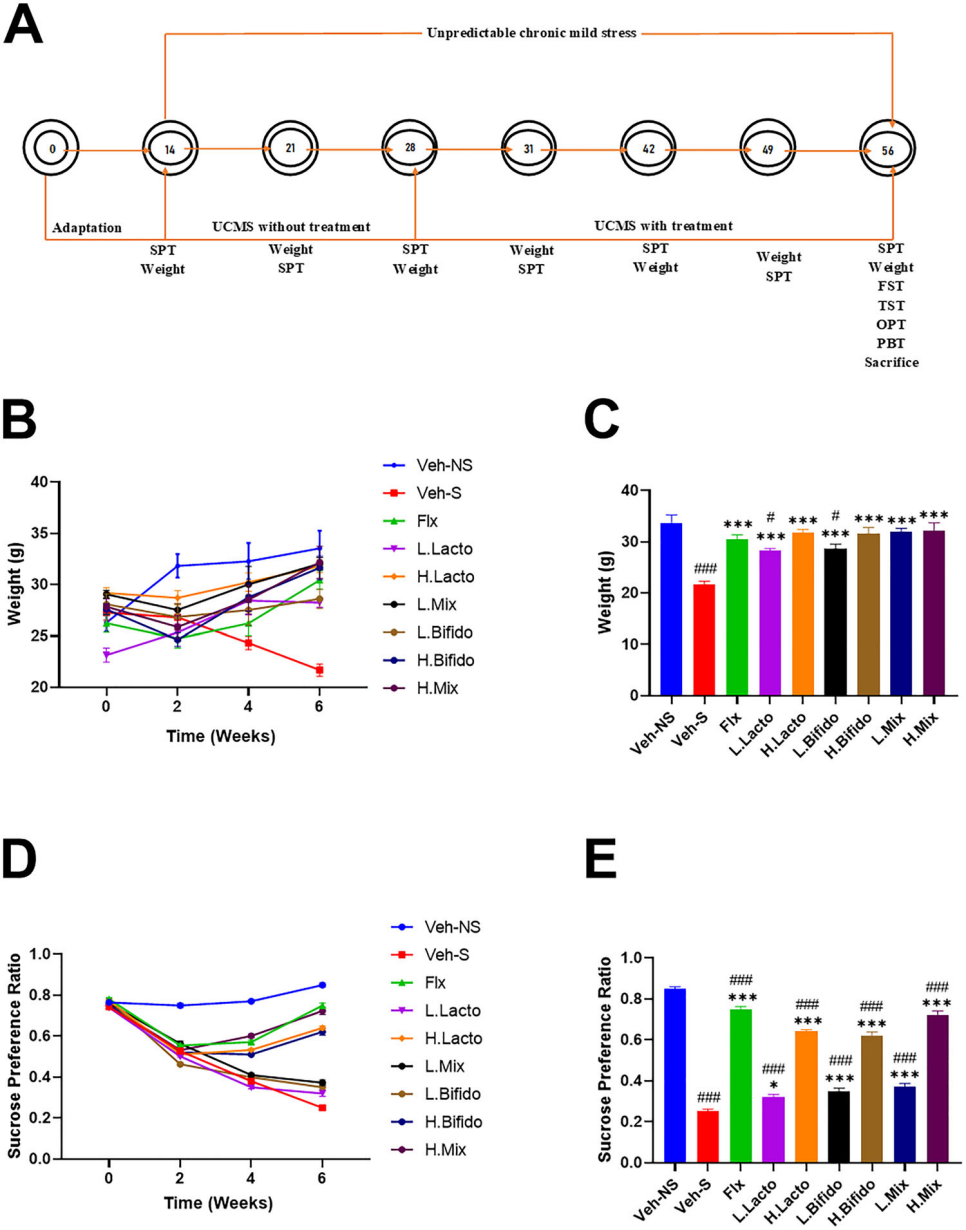
(A) Chronic unpredictable mild stress (UCMS) study scheme and timeline in days. SPT‐sucrose preference test, FST‐force swim test, TST‐tail suspension test, OPT‐open field test, PBT‐pain‐like behavior test. The numbers in circles represent days. (B) Effect of formulations on weight or (D) sucrose preference test over the six week experimental period. (C) Animal weights and (E) sucrose preference ratio at week 6 showing concentration‐dependent benefits of *Lactobacillus (*H.Lacto, L.Lacto) and *Bifidobacterium (*H.Bifido, L.Bifido) similar to benefits conferred by fluoxetine (Flx). Data are represented as mean ± SEM. (B and D) (two‐way ANOVA followed by Bonferroni post hoc test); (C and E) (one‐way ANOVA followed by Tukey's multiple comparison test). #P < 0.05, ### P < 0.001 when compared to control group and * P < 0.05, *** P < 0.001 when compared to the stress group.

Chronic unpredictable mild stress (CUMS) is an established behavioral model used to study depression and associated behavioral deficits such as anxiety and anhedonia (Zhu et al. 2014). Over a 6‐week period, the mice were exposed to six different stressors as part of the CUMS protocol: food deprivation, water deprivation, reversed light/dark cycle, wet bedding, cage tilting at 45 °, and no bedding. One stressor was randomly applied each day to ensure predictability. The first two weeks of CUMS were without therapeutic intervention, whereas the final four weeks consisted of CUMS and group‐specific therapeutic interventions. Controls were maintained under normal housing conditions without stress for 6 weeks. The primary variables measured were weight and sucrose preference at four different time points: before CUMS (week 0), before therapeutic intervention (week 2), intervention midway point (week 4), and at the end of the experimental period (week 6). Additional behavioral tests were performed at week 6 to measure depression and memory deficits. A schematic of the experimental protocol is shown in Figure 1A.

### 2.5 Sucrose Preference Test (SPT)

Sucrose preference tests were performed as described by Willner et al. (1987) with minor modifications. To measure sucrose preference, mice were adapted to sucrose for two days prior to deprivation of water and food for 24 h. Subsequently, they had free access to two bottles containing 100 mL of sucrose solution (1% w/v) or 100 mL of water placed in individual cages. After an hour, the volumes of sucrose solution and water consumed were recorded, and sucrose preference was calculated as follows:

$$\mathrm{Sucrose}\;\mathrm{Preference}=\frac{sucroseintake}{sucroseintake+waterintake}$$

### 2.6 Open Field Test (OFT)

The open field test was performed as described by Shukkoor et al. (2016) to assess anxiety and locomotor behavior. The open field apparatus was a 100 cm × 100 cm × 40 cm box with 25 equal square sections separated by lines on the floor of the box. The top of the enclosure was uncovered to observe the movement of the animal using a video camera. Each mouse was placed in the center of the field and allowed unlimited access to any location for six minutes. After removing the mice from the field, the enclosure was cleaned with 70% ethanol to remove the odor cues. Mouse activity during the open field test was recorded for subsequent analysis using a video camera (AI triple camera (13 MP), Tecno Spark4, Tecno Mobile).

### 2.7 Force Swim Test (FST)

The FST was used to evaluate the state of helplessness in stressed mice after the CUMS protocol as described by Porsolt et al. (2008). Briefly, 60 min prior to the FST, mice were moved to the test room after which they were placed individually in a plastic container (total volume: 1000 mL, 21 cm height, and 12 cm diameter) filled to a depth of 10 cm with water (23°C±2°C). Each mouse was exposed to a test session for 6 min, and the entire experimental process was recorded for subsequent analysis (AI triple camera (13 MP), Tecno Spark4, Tecno Mobile). After the session, mice were partially dried with a towel and allowed to fully dry in their home cages without any further intervention. Immobility time was measured as the amount of time the mice remained still while floating with their heads above the water without making any effort to move. The swimming and climbing times were recorded.

### 2.8 Tail Suspension Test (TST)

The tail suspension test was performed 24 h after the forced swim test. TST was performed as previously outlined by Thierry et al. (1986), with slight modifications. The mice were allowed to acclimatize to room conditions for an hour prior to the experiment. The mice were suspended by the tail from a metal rod mounted 30 cm above the surface by fastening the tail to the rod with adhesive tape. Mouse activity was recorded using a video camera (AI triple camera (13 MP), Tecno Spark4, Tecno Mobile) for 6 min and subsequently analyzed offline for immobility, curling, and swinging behavior.

### 2.9 Hot Plate Test

The mice were allowed to acclimatize to room conditions for an hour prior to the experiment. Each mouse was placed on an unrestrained metal surface heated to 54°C, which was enclosed around it but had an open top. The latency to nocifensive actions, including forepaw withdrawal or licking, hind paw withdrawal or licking, stamping, hunching down, vocalization, and jumping, was recorded (Espejo and Mir 1993). After a predetermined cutoff time of 20 s, the animal was removed from the hot plate if no nocifensive responses were observed; thus, tissue injury may be prevented. This test was performed 24 h after the tail suspension test.

### 2.10 Data Analysis

Results are expressed as mean ± standard error of the mean (SEM). Statistical analysis was carried out by one‐way analysis of variance (ANOVA) followed by Tukey's multiple comparison, or a two‐way analysis of variance (ANOVA) followed by Bonferroni post hoc test using GraphPad Prism version 8 (GraphPad Software, Inc., La Jolla, CA, USA). Differences were considered statistically significant at P < 0.05.

## 3 Results

### 3.1 Effects of Probiotic Administration on Body Weight

Body weight was measured at four time points (week 0, week 2, week 4, and week 6) throughout the study, with significant differences observed over time (two‐way ANOVA, F(1.3, 71) = 79; p < 0.001; Figure 1B). Between week 0 and week 2, the control group recorded an increase in body weight (from 26.3±0.9 g to 31.8±1.1 g). Body weight tended to decrease after 2 weeks of CUMS, however, this decrease was significant in only a subset of groups (Figure 1B; Flx: from 26.2±0.9 g to 24.8±0.9 g, H.Bifido: from 27.6±0.5 g to 24.6±0.7 g, L.Mix: from 29.1±0.4 g to 27.5±0.6 g, H.Mix: from 27.8±0.8 g to 25.9±1.1 g). Upon commencement of the therapeutic intervention, the treatment groups gradually began to experience weight gain, while the stress‐only group (Veh‐S) showed no sign of weight gain but further weight loss. Comparing the average weight between the groups at the end of the sixth week revealed significant differences, with the Veh‐S, L.Lacto, and L.Bifido groups having a significantly lower weight in comparison to controls (one‐way ANOVA, F(8,54) = 11.69; p < 0.001) (Figure 1C).

### 3.2 Sucrose Preference Test

Except for the control group, all other groups exhibited a significant decrease in sucrose preference between week 0 and week 2 (Figure 1D; Veh‐S: from 0.74±0.01 to 0.53±0.01, Flx: from 0.78±0.01 to 0.55±0.01, L.Lacto: from 0.74±0.01 to 0.5±0.01, H.Lacto: from 0.77±0.01 to 0.51±0.01, L.Bifido: from 0.77±0.01 to 0.46±0.01, H.Bifido: from 0.75±0.01 to 0.52±0.01, L.Mix: from 0.76±0.01 to 0.56±0.01, H.Mix: from 0.75±0.01 to 0.53±0.01; F(1.8, 99) = 1238; p < 0.001). For the stressed L.Lacto, L.Bifido, and L.Mix groups, this decrease continued on through to week 6 (Veh‐S: 0.25±0.01; L.Lacto: 0.32±0.01; L.Bifido: 0.35±0.01; L.Mix: 0.37±0.01). On the other hand, a restoration of the sucrose preference was observed at week 6 for the Fluoxetine (0.75±0.01), H.Lacto (0.64±0.01), H.Bifido (0.62±0.02), and H.Mix groups (0.72±0.02), with scores returning to near pre‐CUMS baseline levels. At the end of the 6th week, all treatment groups had higher sucrose preference scores than the stress group, with the largest effect size observed in the fluoxetine and high probiotic concentration groups (Figure 1E; one‐way ANOVA F(8,54) = 26; p < 0.001).

### 3.3 Effect of Probiotic Administration on Behavioral Measures of Depression and Anxiety

**FIGURE 2:**
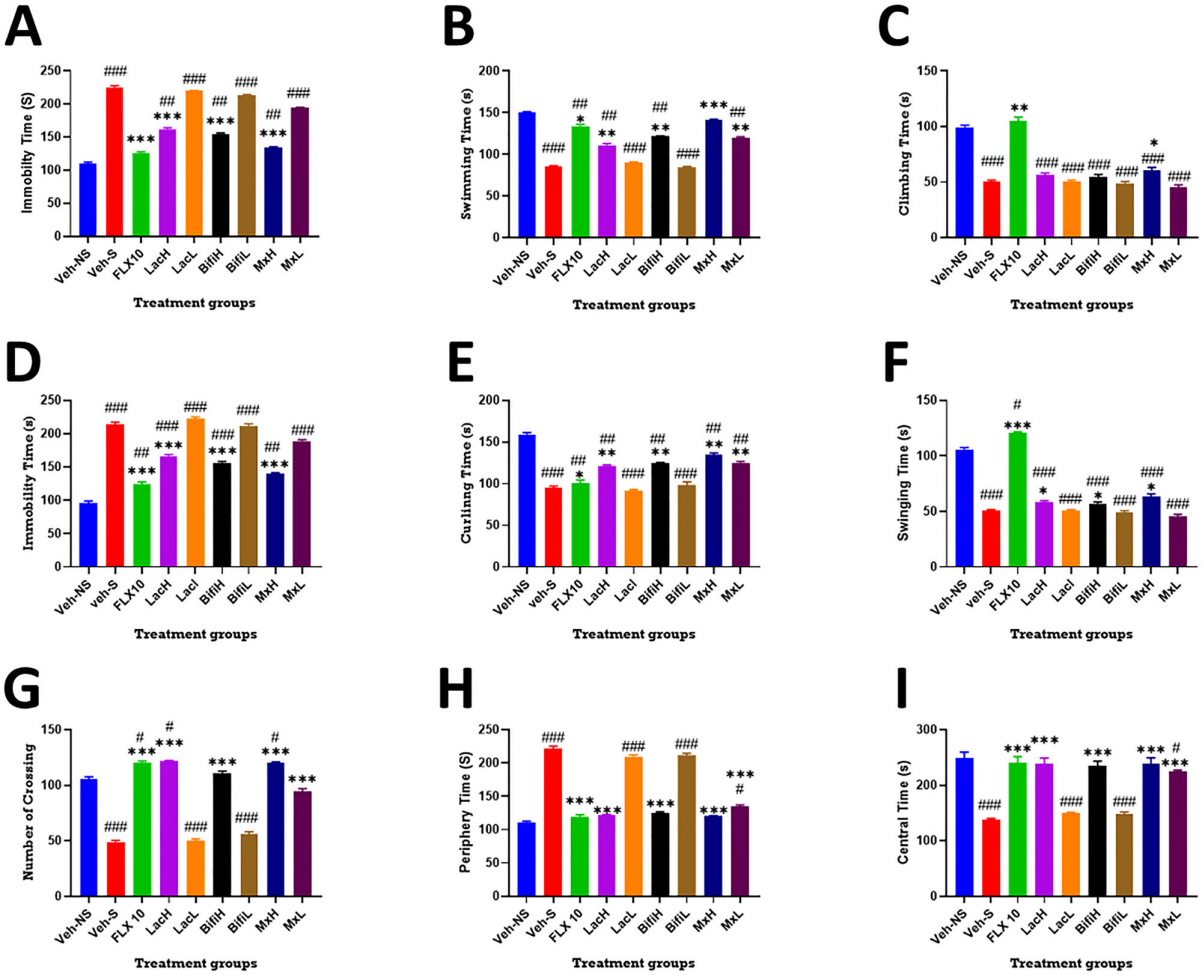
Effect of probiotic administration on behavioral measures of depression, helplessness, and anxiety. (A)–(C) Force swim test: probiotics decreased stress induced immobility (A) Immobility time (s), (B) swimming time (s), and (C) climbing time (s). (D)–(F) Tail suspension test: probiotics reversed stress induced behavioral deficits but not to the same degree as fluoxetine (D); Immobility time (s), (E) curling time (s), and (F) swinging time (s). (G)–(I) Open field test: probiotics protected against stress‐induced deficits in exploratory behavior. (G) Number of crossings, (H) periphery time (s), (I) c time (s). Data are presented as mean ± SEM. One‐way ANOVA followed by Tukey's multiple comparison test) # P < 0.05, ## P < 0.01, ### P < 0.001, when compared to the control group (Veh‐NS); *P < 0.05, ** P < 0.01, *** P < 0.001 when compared to the stress group (Veh‐S).

The FST results showed significant differences in immobility time (Figure 2A, F(8,54) = 927; p < 0.001), swimming time (Figure 2B, F(8,54) = 167; p < 0.001), and climbing time (Figure 2C, F(8,54) = 301; p < 0.001) between the groups. When compared to the controls, the stressed group (Veh‐S) spent more time immobile (Veh‐NS: 110.1±1.8s versus Veh‐S: 225±2.4s), and less time swimming (Veh‐NS: 150.1±1.1s versus Veh‐S: 85±1.3s) or climbing (Veh‐NS: 99.6±1.8s versus Veh‐S: 50.4±1.3s). This stress‐induced deficit in immobility and swimming time was reversed to varying degrees by fluoxetine and probiotic therapeutic intervention. However, deficits in climbing time were reversed by only fluoxetine (140.3±0.81 s) or high doses of the combination therapy (H.Mix: 60.6±2.6 s) (Figure 2C).

Similar to the FST, the TST revealed significant differences in the immobility time (Figure 2D, F(8,54) = 283.2; p < 0.001), swinging time (Figure 2E, F(8,54) = 273.8); p < 0.001), and curling time (Figure 2F, F(8,54) = 80.3; p < 0.001) between the groups. Compared to the controls, the stress only group spent significantly more time immobile (Veh‐NS: 96±2.8s versus Veh‐S: 214±3.1s) and less time engaging in curling (Veh‐NS: 159±2.2s versus Veh‐S: 94.9±2.1s) or swinging behavior (Veh‐NS: 105.7±1.9s versus Veh‐S: 50.4±1.3s). Low doses of monotherapy had no effect on the stress‐induced deficits. In comparison to the stress group, immobility time was reduced by fluoxetine (Flx: 124.3±3.4s), combination therapy (L.Mix: 189±1.9s; H.Mix: 140±1.3s) or high doses of monotherapy (H.Lacto: 166±2.4s; H.Bifido: 156±1.9s; Figure 2D). Deficits in curling behaviour were not reversed by fluoxetine (Flx: 100.3±4.1s) although this was achieved by the combination therapy (L.Mix: 125±1.7s; H.Mix: 135±1.8s) or a high doses of monotherapy (H.Lacto: 122±0.8s; H.Bifido: 124±1.5s; Figure 2E). However, deficits in swinging time were reversed to various degrees by fluoxetine (Flx: 120.6±1.1s), a high dose of lactobacillus (H.Lacto: 58.1±1.7s) or high dose of combination therapy (H.Mix: 63.4±2.2s).

Locomotor and anxiety‐like behaviors were assessed using the open‐field test (Figures 2G–I). The results showed a significant difference between the treated groups in the number of squares crossed (Figure 2G one‐way ANOVA F(8,54) = 33.76; p < 0.001), time spent in the periphery (Figure 2H one‐way ANOVA F(8,54) = 42.8.4; p < 0.001), and time spent in the central zones of the arena (Figure 2I one‐way ANOVA F(8,54) = 34.07; p < 0.001). Compared to the controls (Veh‐NS), the stress only group (Veh‐S) crossed less lines (Veh‐NS: 106±2 versus Veh‐S: 49±2), spent more time in the periphery (Veh‐NS: 111±1.7 s versus Veh‐S: 222±3.1 s), and less time in the central zones (Veh‐NS: 248±11.4 s versus Veh‐S: 138±1.9 s). Combination therapy, high doses of monotherapy, and fluoxetine reversed all stress‐induced deficits in the OFT (Figures 2G–I).

### 3.4 Pain Score

**FIGURE 3:**
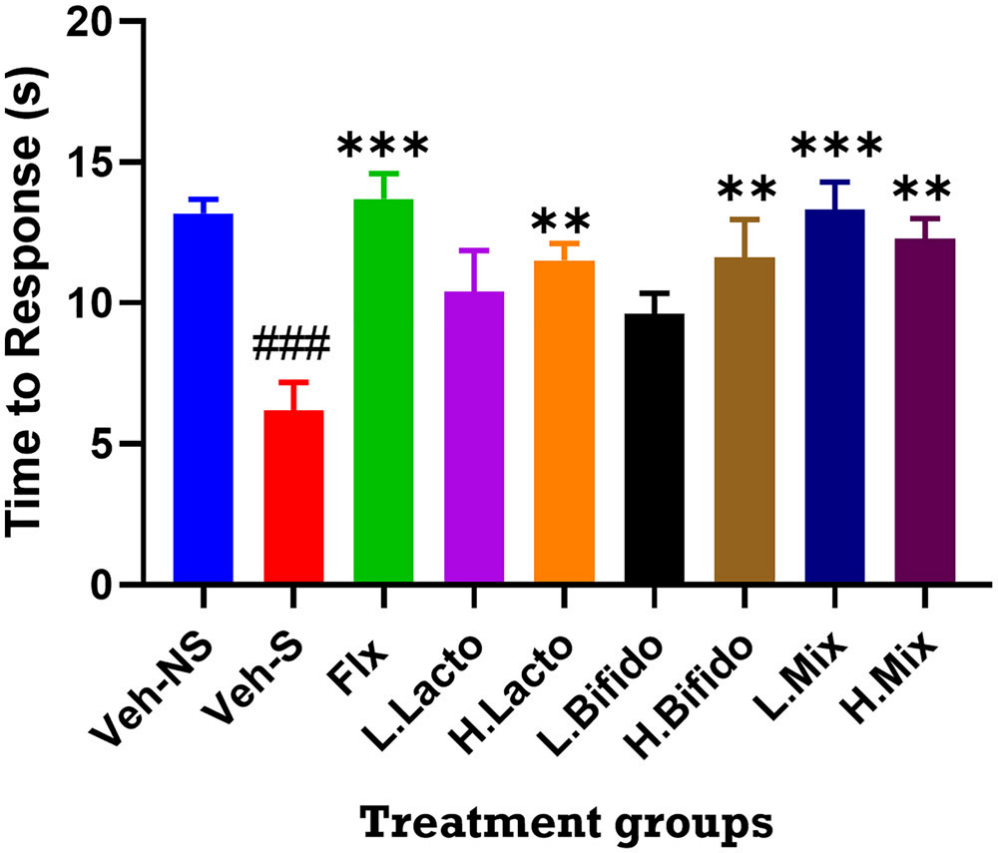
Effect of probiotic administration on pain response time (s). Probiotics protected against stress‐induced increased pain sensitivity to levels similar those two of fluoxetine (flx). Data are presented as mean ± SEM. **P < 0.01, *** P < 0.001 when compared to the vehicle stress group (Veh‐S) and ### P < 0.001when compared to the vehicle control group (Veh‐NS) (one‐way ANOVA followed by Tukey's multiple comparison test).

One‐way ANOVA followed by Tukey's multiple comparison test revealed a significant difference in heat‐induced nociceptive responses (Figure 3, F(8,54) = 5.891; p < 0.001). The stress group was significantly more sensitive to heat stimuli as compared to controls (Veh‐NS: 13.2±0.5s; Veh‐S: 6.2±1s). Aside from groups receiving low doses of Lactobacillus (10.4±1.5s) or Bifidobacteria (9.6±0.7s), all other therapeutic interventions significantly reversed this stress induced sensitivity to heat stimuli (Flx: 13.7±0.9 s; H.Lacto: 11.5±0.6 s; H.Bifido: 11.6±1.4 s; L.Mix: 13.3±1s; H.Mix: 12.3±0.7 s).

## 4 Discussion

This study provides evidence that probiotics *Lactobacillus* and *Bifidobacterium* spp. are capable of reversing depression and anxiety‐like behaviors induced by chronic unpredictable mild stress in a murine model. The effects of probiotics were most pronounced at high doses, with combination therapy being more beneficial than monotherapy, at a level similar to the effect of fluoxetine.

Several criteria have been used to assess depression in animal and human models. Body weight loss or suppression of weight gain and anhedonia are the two main criteria (Zhu et al. 2014; Serchov et al. 2016; Shukkoor et al. 2016). In this study, the first two weeks of CUMS induced weight loss and anhedonia in all test groups (Figures 1B and D), providing sufficient evidence that the stress protocol successfully induced a depression‐like phenotype (Pałucha‐Poniewiera et al. 2020; Park et al. 2018; Taksande et al. 2013). While weight loss may be a consequence of altered energy metabolism (Zhu et al. 2014) or decreased appetite (He et al. 2020b), anhedonia is attributed to neurotransmitter dysfunction and altered activity in specific brain regions (Gorwood 2008 **)**. Weight loss and anhedonia were observed at levels comparable to those reported previously (He et al. 2020b; Liao et al. 2020; Thakare et al. 2018; Zhu et al. 2014).

Between weeks 2 and 6, the stress‐only group (Veh‐S) continued to lose weight; however, all treatment groups gained weight. The least significant weight gain was observed in the low‐dose monotherapy groups, whereas the combination and high‐dose monotherapy groups all had weights that were indistinguishable from controls at week 6. As a positive control, fluoxetine (10 mg/kg) had effects in line with previous reports showing that it reverses CUMS‐induced weight loss (Ji et al. 2020; Shukkoor et al. 2016; Zhu et al. 2014). Variations in the reversal of weight loss between treatment groups were the first piece of evidence suggesting that probiotics reversed stress‐induced phenotypes in a dose‐dependent manner. This was supported by the sucrose preference test at week 6, as reversal of anhedonia followed the same trend and was most significant at high doses of probiotics (mono‐ or combination therapy), to a level similar to the effects of fluoxetine.

The reversal of anhedonia by high probiotic doses to near baseline levels was not immediate, but rather had a time course of action, similar to fluoxetine, which usually requires 2–6 weeks to take effect (Ji et al. 2020; Shukkoor et al. 2016; Zhu et al. 2014). The time required for the prolonged ingestion of probiotics to significantly change the gut microbiome explains the time‐dependent nature of probiotic intervention. Available evidence suggests that oxidative stress due to adverse environmental conditions (which the CUMS mimics) can lead to the depletion of ATP, and this, alongside decreased activity in the reward circuitry, may contribute to depressive‐like behavior (Crema et al. 2010; Tiwari et al. 2002; Gorwood 2008). Potential antidepressant‐like effects could then require the release of endogenous ATP in astrocytes or the increased availability of neurotransmitters such as serotonin (Crema et al. 2010; Gorwood 2008). *Lactobacillus and Bifidobacterium* strains have been shown to enhance serotonergic signaling and increase BDNF and GABA receptors in the amygdala and hippocampus (Johnson and Foster 2018; Tian et al. 2019; Wu et al. 2022). Another benefit of Lactobacillus strains is the enrichment of bifidobacteria (Zhou et al. 2022); therefore, this could account for the positive effect of combination therapy at low doses, where monotherapy had no effect. This is important because it means that individuals who are immunocompromised or cannot have too much exposure to one bacterial strain can still benefit from the MxL treatment.

Stress changes the intestinal microbiota of animals, resulting in a decrease in the abundance of *bifidobacteria* and *lactobacilli* in the gut (O'Mahony et al. 2009). Bifidobacteria have been observed to be susceptible to the negative effects of emotional stress (Goncharova et al. 1981). In light of the possibility that probiotic bacteria therapy might reduce the negative effects of stress, it can be concluded that the exogenous administration of probiotics would have at least stabilized the bifidobacterial presence if not enhanced it (Gareau et al. 2007). Further work, such as gut microbial diversity and metabolite profiling, will have to be performed to confirm this.

Beyond anhedonia as measured by the SPT, animal despair studies can measure the intensity of depression induced, and the two widely accepted assays are the forced swim test (FST) and the tail suspension test (TST) (Dhayabaran and Margret 2017; Pahwa and Goel 2019). In stressed mice, the FST is distinguished by a passive phase (immobile behavior) and an active phase (swimming and climbing). Similarly, TST includes both an active phase (swinging or curling) and an inactive phase (immobile behavior). Low doses of probiotic monotherapy did not confer any benefits, whereas combination and high doses of probiotic monotherapy differentially reversed the indicators of helplessness and immobility as measured in the FST and TST. This further supports the dose‐dependent nature of probiotic activity (Jäger et al. 2019). Compared to the vehicle stress group (Veh‐S), probiotics increased active behaviors, such as swimming, swinging, curling, and climbing. In the FST, increased swimming behavior is characteristic of serotonergic antidepressant activity, whereas climbing behavior predominates for drugs acting via noradrenergic pathways (Duman 2010). Conversely, in the TST, serotonergic and noradrenergic pathways underlie the increase in swinging behavior, whereas opioidergic neurotransmission activity is responsible for the increase in curling behavior (Iman et al. 2020).

Based on the interpretation of behavioral evidence, the probiotic mechanism of activity overlaps with that of fluoxetine. There was a decrease in immobility time in both FST and TST and an increase in swinging time in the TST (MxH). Interestingly, an increase in the curing time in the TST was absent in the fluoxetine group. This suggests that, in addition to enhancing serotonergic activity, the beneficial effects of probiotics could be mediated via additional pathways that could be opioidergic or noradrenergic in nature (Liu et al. 2020; Desbonnet et al. 2010; Wu et al. 2022). However, without molecular measures of monoamine levels, inferring the mechanisms of action based on behavioral tests should be performed cautiously. In particular, contrary to its mechanism of action, fluoxetine, which is primarily a selective serotonin reuptake inhibitor with minimal noradrenergic activity (Berrocoso et al. 2013; REF), has a greater effect on climbing behavior than swimming behavior.

It is also worth noting that the antidepressant effect of probiotics was not due to any psychostimulant effect of the formulations. Similar to antidepressants, psychostimulant agents decrease immobility time, leading to false‐positive results (Pahwa and Goel 2019; Shukkoor et al. 2016). To rule out the possibility that probiotic formulations might have a psychostimulant effect on mice, the open field test (OFT) was used to assess locomotor activity in mice. The study's findings demonstrate that the CUMS technique markedly reduced exploratory activity in the anxious vehicle‐stressed group and at low probiotic concentrations. In this study, exploratory activity was restored in increased line crossing and central activity with decreased peripheral activity (Figure 2H) without necessarily increasing overall locomotive activity (Pahwa and Goel 2019; Shukkoor et al. 2016; Park et al. 2018; Thakare et al. 2018). This proves that the antidepressant‐like effects of probiotics shown in the FST are true and that no psychomotor stimulant activity was responsible for the reduced immobility time in the FST.

Increased sensitivity to pain stimuli has been reported alongside depression; therefore, we questioned whether the effects of probiotics extend to reducing pain sensitivity. In response to heat stimuli (hot plate test), there was a significant difference in the high probiotic concentration groups (LacL, BifiH, and MxH), the mixed low concentration group (MxL), and the fluoxetine group (FLX) when compared to the vehicle stressed group (Veh‐S). Probiotics maintained the time it took to respond to pain in the same way as the vehicle's unstressed controls (Veh‐NS). This could be an indication that probiotics contain opioid properties, as hinted at in the TST results, and can be used to manage stress. It has been reported that the probiotic species Lactobacillus and Bifidobacterium improve the metabolism of tryptophan (Agus et al. 2018). These probiotics shift host tryptophan metabolism by inhibiting the kynurenine pathway, thereby increasing serotonin production (Desbonnet et al. 2008; Gao et al. 2020). Lactobacillus and Bifidobacterium probiotics can also increase TPH1 expression, which indirectly increases colonic serotonin production (Hara et al. 2018). These pathways influence (increase) the peripheral serotonin pool, and may therefore lead to pain relief during stress management when probiotics are administered.

## 5 Conclusion

This study shows that probiotics have a dose‐dependent antidepressant‐like effect in mice by reversing anhedonia and weight loss. Probiotics exhibit antidepressant properties by decreasing immobility, enhancing active escape behaviors, and reducing stress‐induced sensitivity to pain. Taking advantage of the synergistic activity of probiotics makes them equally effective at low doses.

## Author Contributions

**Aliu Zanzeh Bankah**: methodology, investigation, writing–original draft, conceptualization, visualization. **Thomas Amatey Tagoe**: conceptualization, methodology, writing–original draft, writing–review and editing, visualization, project administration, resources, supervision, validation, data curation. **Emmanuel Darko**: writing–review and editing, visualization, investigation. **Righteous Agoha**: investigation, writing–review and editing, formal analysis. **Elmer Nayra Ametefe**: conceptualization, methodology, supervision, resources, writing–review and editing, data curation. **Kennedy Kwami Edem Kukuia**: writing–review and editing, supervision, resources, validation. **Samuel Adjei**: conceptualization, resources, supervision, writing–review and editing, validation.

### Peer Review

The peer review history for this article is available at https://publons.com/publon/10.1002/brb3.70564

## Data Availability

The data that support the findings of this study are available from the corresponding author upon reasonable request.
